# Tailoring lipid nanoparticle dimensions through manufacturing processes†

**DOI:** 10.1039/d4pm00128a

**Published:** 2024-09-23

**Authors:** Caitlin McMillan, Amy Druschitz, Stephen Rumbelow, Ankita Borah, Burcu Binici, Zahra Rattray, Yvonne Perrie

**Affiliations:** a University of Strathclyde Glasgow UK yvonne.perrie@strath.ac.uk; b Croda International Plc and Avanti Polar Lipids Alabaster AL USA

## Abstract

Lipid nanoparticles (LNPs), most commonly recognised for their role in COVID-19 mRNA vaccines, are important delivery vehicles for nucleic acid (mRNA, siRNA) therapies. The physicochemical attributes, such as size, nucleic acid encapsulation and electric charge, may have a significant impact on the efficacy of these medicines. In this study, adjustments to aqueous to lipid phase ratios were assessed for their impact on LNP size and other critical quality attributes (CQAs). It was observed that minor adjustments of aqueous-to-organic lipid phase ratios can be used to precisely control the size of ALC-0315-formulated LNPs. This was then used to evaluate the impact of phase ratio and corresponding size ranges on the *in vitro* and *in vivo* expression of these LNPs. In HEK293 cells, larger LNPs led to higher expression of the mRNA cargo within the LNPs, with a linear correlation between size and expression. In THP-1 cells this preference for larger LNPs was observed up to 120 d.nm after which there was a fall in expression. In BALB/c mice, however, LNPs at the lowest phase ratio tested, >120 d.nm, showed reduced expression compared to those of range 60–120 d.nm, within which there was no significant difference between sizes. These results suggest a robustness of LNP expression up to 120 d.nm, larger than those <100 d.nm conventionally used in medicine.

## Abstract 2

LNP particle size control can be achieved through manufacturing processes. Changes in particle size affect *in vitro* efficacy but not *in vivo* potency. Created with BioRender.com.

## Graphical abstract

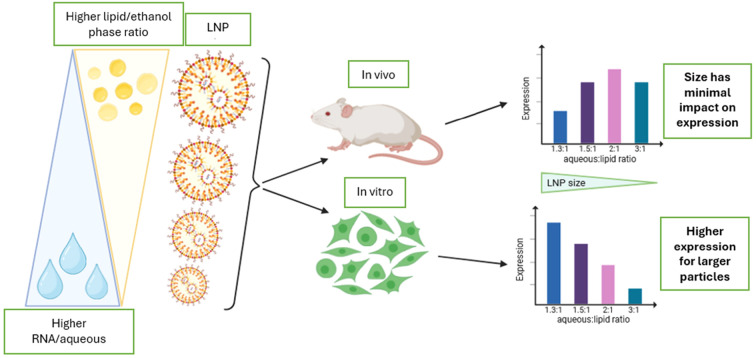

## 1. Introduction

Lipid nanoparticles (LNPs) lie within the family of lipid-based nanoparticles, which also includes liposomes, and other nanoscale lipid complexes. What differentiates LNPs from other lipid-based nanoparticle formats is the presence of a solid core created from nucleic acid complexed to a permanent or ionisable cationic lipid. Thus, LNPs are the vector of choice for the delivery of RNA-based therapeutics and vaccines.^1–3^ The COVID-19 pandemic placed LNPs in the spotlight, hugely increasing interest and recognition of their potential in drug and vaccine delivery, which is evidenced by a doubling of published literature on LNPs in the last 5 years.^4^

Optimising LNP manufacture has emerged as valuable research, especially concerning production speeds, scalability, stability and efficacy of the end product.^5,6^ The growth of this field is driven by challenges faced during the rapid mass production of LNP-based mRNA vaccines during the pandemic. One of the biggest challenges was producing formulations rapidly to meet demand. Furthermore, manufacturing formulation parameters have been widely explored in the field of liposomes,^7,8^ so translating this research to LNPs is valuable. In addition to optimisation of the manufacturing process, testing the robustness of parameters used is vital, as this aids in understanding the formulation, as well as defining the limits within formulations, and expands the knowledge base in nanomedicine formulation strategy. For example, there is evidence that size impacts LNP immunogenicity and mRNA expression, impacting therapeutic efficacy.^3,9,10^ However, the impact varies based on the indication and pharmacokinetics; while larger LNPs (>100 d.nm) may have higher mRNA expression,^10^ there is evidence that smaller LNPs (<100 d nm) are more readily absorbed from the injection site.^9^

Microfluidics is a precise, reproducible, and scalable method for manufacturing LNPs. It has easily adjustable parameters, which can be precisely controlled with relative ease in comparison with other methods of LNP manufacturing.^11^ Parameters, such as mixing speed and flow-rate ratio, can significantly impact the critical quality attributes of the produced LNPs. The flow-rate ratio is equivalent to the ratio of nucleic acid-containing aqueous phase to organic phase dissolved in ethanol. Previous work has shown that higher proportions of organic phase and ethanol increase particle size in liposomes and some formulations of LNPs.^3,7,11,12^ In this study we assess different formulations of LNPs and correlate their *in vitro* and *in vivo* responses, with a specific focus on the LNP mRNA expression.

The aim of this study was to assess whether the phase ratio to size relationship translates to two clinically used LNP formulations, *i.e.* those LNPs formulated with ALC-0315 and SM-102 as the ionisable lipid, and evaluate the impact of phase ratio and LNP particle size on *in vitro* and *in vivo* expression.

## 2. Materials and methods

### 2.1. Materials

1,2-Distearoyl-*sn-glycero*-3-phosphocholine (DSPC) was obtained from Lipoid (Ludwigshafen, Germany). 1,2-Dioleoyl-3-trimethylammoniumpropane (chloride salt) (DOTAP), and 1,2-dimyristoyl-*rac-glycero*-3-methoxypolyethylene glycol-2000 (DMG-PEG 2000) were purchased from Avanti Polar Lipids (Alabaster, AL, USA). Cholesterol, citric acid, sodium citrate tribasic dehydrate, polyadenylic acid (PolyA), 6-(*p*-toluidino)-2-naphthalenesulfonic acid sodium salt, sodium phosphate monobasic (NaH2PO4·H_2_O), and sodium phosphate dibasic (anhydrous) (Na_2_HPO_4_) were acquired from Sigma-Aldrich (St Louis, MO, USA). Phosphate-buffered saline tablets (PBS pH 7.4) were acquired from Oxoid Ltd (Basingstoke, UK). Tris base and ethanol (EtOH) were obtained from Fisher Scientific (Loughborough, UK). Methanol and propan-2-ol (isopropanol, IPA) were purchased from VWR Chemicals (Lutterworth, UK). The ionisable lipids [(4-hydroxybutyl)azanediyl]di(hexane-6,1-diyl) bis(2-hexyldecanoate) (ALC-0315) and 9-heptadecanyl 8-((2-hydroxyethyl)[6-oxo-6-(undecyloxy)-hexyl]amino)octanoate (SM-102), and the PEG-lipid 2-[(polyethylene glycol)-2000]-*N*,*N*-ditetradecylacetamide, 1,2-distearoyl-*sn-glycero*-3-phosphocholine (ALC-0159), were purchased from BroadPharm (San Diego, CA, USA). Minimum Essential Medium, Trypsin-EDTA (0.05%), PBS, pH 7.4 solution, Quant-it™ RiboGreen RNA Assay Kit, RiboGreen RNA Reagent, and RediPlate™ 96 RiboGreen™ RNA Quantitation Kit were purchased from Fisher Scientific UK Ltd (Renfrew, UK). EZ Cap™ Firefly Luciferase mRNA was purchased from ApexBio Technology (Houston, TX, USA). mGreenLantern was provided by CPI *via* the Intracellular Drug Delivery Centre (IDDC) research programme. ONE-Glo™ Luciferase Assay System was acquired from Promega UK (Southampton, UK). All solvents and other chemicals were of analytical grade, and Milli-Q-water was provided by an in-house system.

### 2.2. Aqueous and organic phase preparation

The aqueous phase containing mRNA and the organic phase were prepared separately before microfluidics. The aqueous phase was prepared using 50 mM citrate buffer (pH 4), dissolving PolyA, or Firefly Luciferase (FLuc) mRNA for *in vitro* and *in vivo* experiments, at mRNA concentrations required to maintain a nitrogen-to-phosphate (N/P) ratio of 6, the molar ratio of ionisable lipid containing positively charged amines (N) to the mRNA containing negatively charged phosphates (P). Therefore, the concentrations were adjusted for different aqueous : organic phase ratios and when different lipids or mRNA were used, to maintain this N/P ratio.

The lipid molar ratio of the LNPs was 10 : 38.5 : 50 : 1.5% (structural lipid : cholesterol : ionizable lipid : PEGylated lipid). This led to the following combinations utilised in the experiments: (1) DSPC : Chol : ALC-0315 : DMG-PEG2000, (2) DSPC : Chol : ALC-0315 : ALC-PEG and (3) DSPC : Chol : SM-102 : DMG-PEG2000. The lipid stocks were prepared and stored at concentrations of 5–20 mg mL^−1^, dissolving each lipid in ethanol. Unless otherwise stated, the organic phase injected into the microfluidic device has a concentration of 5 mg mL^−1^.

### 2.3. Manufacture of the LNPs using microfluidics

The NanoAssemblr benchtop (Precision Nanosystems Inc., Vancouver, Canada) was used in all experiments in this paper. The ethanol-based organic phase and the citrate buffer-based aqueous phase containing mRNA in the form of PolyA or FLuc mRNA were fed through syringes into a microfluidics chip with a herringbone mixer design. The phase ratio (aqueous : organic phase) was adjusted using the computer interface of the machine, varying between the limits of 1 : 1–3 : 1 aqueous : organic phase, respectively.

### 2.4. Purification methods

For purification by dialysis buffer exchange, the LNP sample in the original ethanol/citrate buffer was exchanged into 200 mL of PBS (pH 7.4) for every mL of sample. Samples manufactured with PolyA as the internal nucleic acid and mRNA samples for *in vitro* experiments were dialysed for 1 hour at ambient temperature, and for 24 hours at 4 °C for *in vivo* studies. Spin columns of 100 kDa MWCO were used for centrifugation and purification of LNPs alongside dialysis in optimisation experiments. LNPs were diluted 40× in PBS. The samples were spun at 2000*g* at 4 °C for 20–40 minutes (time varying with formulation) until the sample reached the original pre-purification volume.

### 2.5. Measuring critical quality attributes (CQAs)

#### 2.5.1. Physicochemical

Size (*Z*-average diameter), polydispersity index (PDI) and zeta potential were measured using the Malvern Panalytical Zetasizer ZS and Zetasizer Ultra, using the Zetasizer software for data acquisition. Size and PDI were measured *via* dynamic light scattering (DLS), using a measurement angle of 173° backscatter, and zeta potential was measured using voltage measurements in a folded capillary cell. Each of these measurements was performed in triplicate for each experimental repeat (nine measurements for each experiment). Post-purification, each sample was prepared for measurement at a lipid concentration of 0.1 mg mL^−1^ in PBS (for size/PDI) or deionised water (for zeta potential) to achieve attenuation values between 7 and 8. Pre-purification measurements for size and PDI were performed with citrate buffer as the diluent. ZEN0040 disposable micro-cuvettes were used for size and polydispersity, and DTS1070 folded capillary zeta cells were used for zeta potential measurements, adding 400 μL and 1000 μL of diluted LNPs, respectively. The material refractive index and absorption were 1.45 and 0.001, respectively. The dispersant refractive index and absorption were 1.33 and 0.8872 cP, respectively for water, 1.335 and 1.02 cP, respectively for PBS, and 1.47 and 1.28 cP for citrate buffer.

Nanoparticle tracking analysis was performed on samples post-purification using the Malvern Panalytical NanoSight Pro (Malvern Panalytical, Malvern, Worcestershire, UK) and the NS Xplorer software was used for data analysis. Nanoparticle size distribution and estimated particle concentration were measured using a 488 nm laser block and videos were captured using a high sensitivity sCMOS camera with a light-scatter filter. Samples were diluted to 0.05–0.1 μg mL^−1^ and analysed under constant flow (1–1.5 μL min^−1^) using a syringe driver, with the temperature set to 20 °C. Five 11.5 s videos were captured for each sample and optimised using the automated optimal settings employed by the software. Between 2700 and 5100 particle trajectories (valid tracks) were analysed depending on the sample to gather the NTA data. These values were 2774, 3623, 4976 and 5056 for the aqueous : organic phase ratios of 1.3 : 1, 1.5 : 1, 2 : 1 and 3 : 1, respectively, which equate to the total number of particles measured per sample (Table S1†).

#### 2.5.2. mRNA content

Encapsulation efficiency and mRNA recovery were measured using the RiboGreen™ mRNA quantification assay kit according to the manufacturer's recommendations. Tris-EDTA (TE) buffer was used as the diluent in all steps of this assay. The fluorescent dye was used to quantify mRNA in the presence and absence of 1% Triton, for lysis of the LNPs. Two RNA standard curves were used to quantify mRNA. The fluorescence signal was read using the POLARstar Omega and GloMax plate readers using 475–485 nm excitation and 525 nm emission wavelengths.

When analysing the results, the total RNA for each sample from the Triton wells and free (unentrapped) RNA from the TE-only wells were obtained. The following equations were used to calculate encapsulation efficiency (EE%) and mRNA recovery as follows:

1 EE% = 100 × (CT − CF)/CT

2 mRNA recovery (%) = 100 × CT/CI

where CT = total RNA concentration (based on results from the wells prepared with Triton-TE buffer), CF = free, unentrapped RNA concentration (based on results from the wells prepared with TE buffer), and CI = RNA inputted into the experiment, the theoretical concentration of RNA.

#### 2.5.3. Cryogenic transmission electron microscopy (cryo-TEM)

Lipid nanoparticle samples were prepared as previously stated and maintained at manufacturing concentrations in pH 7.4 PBS. 3 μL of each sample was blotted using an FEI Vitrobot (Thermo Fisher Scientific, UK) for 3 seconds and 5 force onto a 300-mesh lacey carbon-coated copper grid, then immediately vitrified in liquid ethane, cooled by liquid nitrogen, and held in a cryo-specimen holder (Gatan Elsa) before transfer to the TEM. LNP morphology was observed and captured at liquid nitrogen temperature using a Jeol JEM-F-200 microscope (Jeol, Japan) at 200 kV.

#### 2.5.4. *In vitro* expression

LNP FLuc mRNA expression was analysed in HEK293, THP-1 and BMM (bone marrow-derived macrophages) cells using firefly luciferase as a bioluminescent reporter. Cells were cultured in minimal essential medium (MEM) (for HEK-293 cells) or RPMI 1640 (for THP-1 and BMM cells), supplemented with 10% foetal bovine serum (FBS), 1% penicillin/streptomycin and 1% sodium pyruvate. Cells were added to a 96-well white/clear bottom plate, at a concentration of 10 000 cells per well in media and cultured for 72 hours, after which LNPs were added. LNPs were formulated as above, with EZ Cap™ Firefly Luciferase mRNA as the internal RNA. LNP formulations were added to the cells, replacing the growth media, and diluted in media at total mRNA concentrations of 2 μg mL^−1^, 1 μg mL^−1^, 0.5 μg mL^−1^ and 0.25 μg mL^−1^, using three wells per concentration and leaving three wells free from LNPs (control wells). Cells were incubated with LNPs for 24 hours prior to the addition of the luciferase reagent. The luciferase reagent, prepared as per the manufacturer's instructions, was added to each well of 100 μL of media and cells containing LNPs from the previous day, creating a total volume of 200 μL, mixed thoroughly and left to incubate for 3 minutes. The luminescence of the plates was read using the GloMax plate reader (Promega, UK).

LNP FLuc mGreenLantern expression was analysed in HEK293, THP-1 and BMM (bone marrow-derived macrophages) cells using Green Lantern mRNA for taking fluorescence images of LNP expression. Cells were cultured as above in 6-well plates and transfected with LNPs at a total mRNA concentration of 2 μg mL^−1^. Cells were incubated with LNPs for 24 hours prior to imaging using the GFP filter on the EVOS M5000 microscope (Thermo Fisher Scientific, UK). The total fluorescence of these images was quantified using ImageJ, using 5 images per sample.

#### 2.5.5. *In vivo* expression

LNP expression was analysed in BALB/c mice using firefly luciferase as a bioluminescent reporter. These LNPs were manufactured incorporating 1% DiR dye in the organic phase, to confirm successful administration of the LNPs at the injection site and with EZ Cap™ Firefly Luciferase mRNA as the internal RNA. Three BALB/c mice were injected with each formulation in each study (*N* = 3 per formulation, 9 mice per formulation). Each mouse was injected intramuscularly (I.M.) with (50 μL in each quadricep) 0.1 mg mL^−1^ mRNA LNPs prior to anaesthesia and imaging. Imaging was performed at four time points after LNP injection: 0, 6, 24 and 48 hours. The mice were anaesthetised and maintained under anaesthesia using isoflurane before imaging using the IVIS® Spectrum and the associated Living Image® software (PerkinElmer, Buckinghamshire, UK). For confirming successful I.M. injection of the LNPs, the presence of DiR was detected using the fluorescence setting, at wavelengths of 710 nm excitation and 780 nm emission. For the FLuc mRNA expression the mice were firstly injected subcutaneously with 30 mg mL^−1^d-luciferin solution (5 μL per gram of body weight, corresponding to 150 mg kg^−1^). This SC injection was repeated at each of the four time points, and these mice were imaged 10 minutes after injection using the luminescence setting. After the 48 hour time point the mice were terminated whilst still under anaesthesia using a schedule 1 method. The images were collated and normalised, and the fluorescence and bioluminescence signals were quantified by selecting regions of interest in the software.

#### 2.5.6. p*K*_a_ determination

The effective p*K*_a_ of the ionisable lipid ALC-0315 within the formulation was determined using 6-(*p*-toluidino)-2-naphthalenesulfonic acid (TNS) as a fluorescent marker for positively charged particles, adapted from Tanaka *et al.*'s method.^13^ The LNPs were titrated in buffers from pH 3–9 and the pH at which 50% of the maximum fluorescence signal was observed was taken as the p*K*_a_. ALC-0315 LNPs were manufactured and diluted to a concentration of 0.5 mM. Buffers of pH 3, 4, 4.5, 5, 5.5, 6, 6.5, 7, 7.5, 8, 8.5 and 9 were prepared at a concentration of 20 mM. Citrate buffer was used for pH 3–6, sodium dihydrogen phosphate buffer was used for pH 6.5–8, and Tris buffer was used for pH 8.5 and 9. TNS was prepared by dissolving 6-(*p*-toluidino)-2-naphthalenesulfonic acid sodium salt in water, to a concentration of 0.6 mM. 186 μL of each buffer was added to the appropriate wells, followed by the addition of 12 μL of LNP solutions, and subsequent addition of TNS solution (2 μL). The fluorescence of TNS was measured using a POLARstar Omega plate reader at 355 nm excitation and 460 nm emission wavelengths.

### 2.6. Statistical analysis

The mean and standard deviation were calculated for all experiments, performed in independent triplicate batches unless otherwise stated, with each batch manufactured on a different day to account for inter-day variation. For hydrodynamic size, polydispersity, and zeta potential, three measurements were taken for each batch and purification status; therefore these groups for statistical analysis were *n* = 9. For statistical comparison of groups, one way (for one independent variable) or two/three-way (for two and three independent variables, respectively) analysis of variance (ANOVA) was performed with Tukey's HSD *post-hoc* for pairwise comparisons. Unpaired *t*-tests were used when comparing just two groups, and paired *t*-tests were used for comparing the same group/batch over time or treatment. In the graphs, statistical significance is indicated by individual *p* values for *p* < 0.05, whereas (ns) denotes no statistical significance.

## 3. Results and discussion

### 3.1. The effect of altering manufacturing parameters on the physicochemical attributes of lipid nanoparticles (LNPs)

To confirm that one hour of dialysis was sufficient for LNP sample purification, samples manufactured at an aqueous-to-organic phase ratio of 3 : 1 were purified by both dialysis and spin columns (Table 1). This showed that although spin columns slightly reduced the size of these LNPs, the other CQAs were unaffected by the differing purification methods. It was necessary to use dialysis for the purification of LNPs in the upper size range. Based on these results, dialysis was used as the purification method for all subsequent samples and extended to 24 hours for samples *in vivo* to optimise buffer exchange.

**Table 1:** Comparison of different purification methods. Dialysis was performed for 1 hour. 100 kDa spin columns were used for centrifugation of LNPs diluted 40× in PBS back to the original sample volume. Each measurement is a mean of three independent batches with error bars showing ±SD. The significance is indicated by (*) *p* < 0.05

Purification status	Size (nm)	PDI	Zeta potential (mV)	EE (%)	mRNA recovery (%)
Before purification	89 ± 13	0.15 ± 0.04	n/a	n/a	n/a
Dialysis	88 ± 10	0.18 ± 0.03	−1.4 ± 0.3	91 ± 2	72 ± 7
Spin column	72 ± 5*	0.12 ± 0.04	−0.4 ± 1.3	95 ± 1	86 ± 6

#### 3.1.1. The impact of aqueous : organic phase ratio across different LNP formulations

**Fig. 1:**
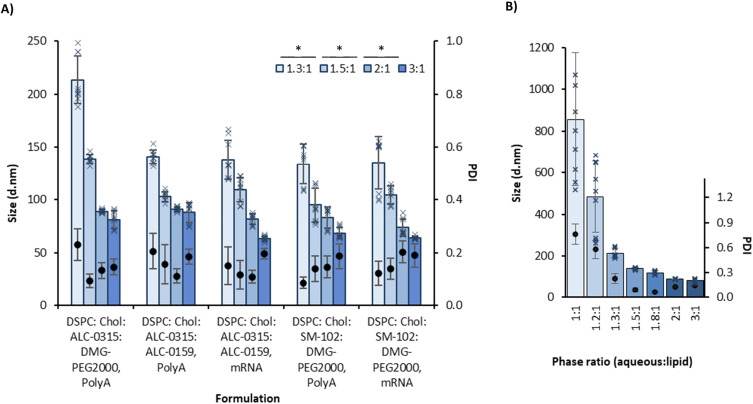
The effect of aqueous : organic phase ratio on LNP size across different lipid combinations and nucleic acid cargos. (A) Comparing phase ratios of 1.3, 1.5, 2 and 3 (:1) in five LNP formulations. (B) Examining a wider range of phase ratios in the first formulation (ALC-0315 : DMG-PEG2000). The sizes are shown as columns, with individual measurements shown as crosses and the polydispersity indices (PDI) are shown as solid circles. The LNPs were manufactured with an N/P ratio of 6 from 5 mg mL^−1^ lipid stock and purified by dialysis in pH 7.4 PBS for 1 hour. Each measurement is a mean of three independent batches with error bars showing ±SD. The significance is indicated by an asterisk (*) between each of the phase ratios analysed by two-way ANOVA with Tukey's pairwise comparison.

The impact of the proportion of aqueous phase to organic phase on the LNP physicochemical attributes, *i.e.* size, polydispersity, zeta potential and nucleic acid recovery, was evaluated across three different LNP formulations. These were (1) DSPC : Chol : ALC-0315 : DMG-PEG2000, (2) DSPC : Chol : ALC-0315 : ALC-0159 and (3) DSPC : Chol : SM-102 : DMG-PEG2000, each at a molar ratio of 10 : 38.5 : 50 : 1.5 for each of the lipids, respectively. LNPs composed of ALC-0315 as the ionisable lipid and DMG-PEG2000 as the PEGylated lipid, were initially used. Seven different aqueous-to-organic phase ratios were tested, and the results showed a trend of decreasing LNP size as the proportion of aqueous phase to organic phase increased (Fig. 1B). For samples manufactured at ratios of 1 : 1 and 1.2 : 1, the polydispersity was high, with values of 0.58 and 0.76, suggesting a highly heterogeneous population. These more heterogeneous LNP batches also tended to show more variability in terms of zeta potential, encapsulation efficiency (EE%) and mRNA recovery (%) (Table 2). Due to this, the mixing ratios of 1 : 1 and 1.2 : 1 were not further investigated. The ratio of 1.8 : 1 was also not included in future experiments for simplification purposes.

**Table 2:** Physicochemical attributes of each of the tested LNP formulations. The LNPs were manufactured with an N/P of 6 from 5 mg mL^−1^ lipid stocks and purified by dialysis in pH 7.4 PBS for 1 hour. Each measurement is a mean of three independent batches ±standard deviation. EE% = encapsulation efficiency

DSPC:Chol:	Aqueous : organic phase ratio	Zeta potential (mV)	EE%	mRNA recovery (%)
ALC-0315:DMG-PEG2000, PolyA	1 : 1	8.3 ± 8.3	70 ± 14	37 ± 17
	1.2 : 1	6.0 ± 6.8	61 ± 19	68 ± 4
	1.3 : 1	4.6 ± 5.0	75 ± 23	65 ± 10
	1.5 : 1	1.8 ± 3.8	96 ± 1	68 ± 10
	1.8 : 1	2.8 ± 3.0	96 ± 1	79 ± 10
	2 : 1	−1.7 ± 1.3	94 ± 1	93 ± 6
	3 : 1	−2.6 ± 1.2	98 ± 1	91 ± 13
ALC-0315:ALC-0159, PolyA	1.3 : 1	0.4 ± 6.4	94 ± 2	97 ± 4
	1.5 : 1	4.0 ± 3.9	90 ± 7	81 ± 2
	2 : 1	1.4 ± 1.7	89 ± 4	75 ± 8
	3 : 1	−1.4 ± 0.3	91 ± 2	72 ± 7
ALC-0315:ALC-0159, mRNA	1.3 : 1	−1.3 ± 2.2	61 ± 18	82 ± 4
	1.5 : 1	−0.2 ± 2.1	79 ± 7	86 ± 7
	2 : 1	−1.5 ± 1.0	89 ± 2	83 ± 12
	3 : 1	−0.9 ± 1.4	91 ± 2	94 ± 13
SM-102:DMG-PEG2000, PolyA	1.3 : 1	5.6 ± 0.8	97 ± 2	78 ± 5
	1.5 : 1	3.7 ± 3.0	98 ± 1	89 ± 5
	2 : 1	4.6 ± 1.5	99 ± 1	95 ± 8
	3 : 1	3.4 ± 1.2	99 ± 1	87 ± 0
SM-102:DMG-PEG2000, mRNA	1.3 : 1	−0.9 ± 12	95 ± 4	93 ± 11
	1.5 : 1	5.2 ± 1.2	94 ± 5	82 ± 11
	2 : 1	4.8 ± 1.4	96 ± 3	84 ± 11
	3 : 1	3.4 ± 1.5	96 ± 3	100 ± 20

The second LNP formulation tested used ALC-0315 in combination with ALC-0159 as the PEGylated lipid, similar to the Pfizer/BioNTech Comirnaty® mRNA vaccine lipid composition. The aqueous : organic phase ratios tested were 1.3 : 1, 1.5 : 1, 2 : 1 and 3 : 1. The same trend of size decreasing as the aqueous phase increased was observed (Fig. 1A). The PDI remained low through all phase ratios of approximately <0.2, and the zeta potential remained neutral (−5 to 5 mV) (Table 2).

This pattern was repeated with LNPs prepared using SM-102 and DMG-PEG2000 (a mimic of the Moderna Spikevax® lipid composition). These formulations were measured using both PolyA and mRNA as the nucleic acid cargo, both of which gave the same phase ratio–size relationship. A two-way ANOVA with Tukey's *post hoc* analysis found significant differences in size output for all phase ratios, with average sizes being approximately 3 : 1 = 70 d.nm, 2 : 1 = 80 d.nm, 1.5 : 1 = 110 d.nm and 1.3 : 1 = 145 d.nm (aqueous : organic phase) (Fig. 1A). Within the phase ratios tested, the PDI remained low (<0.2), the zeta potential was near neutral and EE% and mRNA recovery% were >60% (Table 2). This correlation between phase ratio and LNP particle size results from the rate of polarity change induced by ethanol dilution with the aqueous phase. Because lipids are amphiphilic, as ethanol is diluted (and polarity increases), the lipids self-assemble to form LNPs. In higher proportions of aqueous phase to ethanol concentrations, the ethanol is diluted faster, the lipids have less opportunity to aggregate, and hence, this drives the formation of smaller LNPs.^14^

**Fig. 2:**
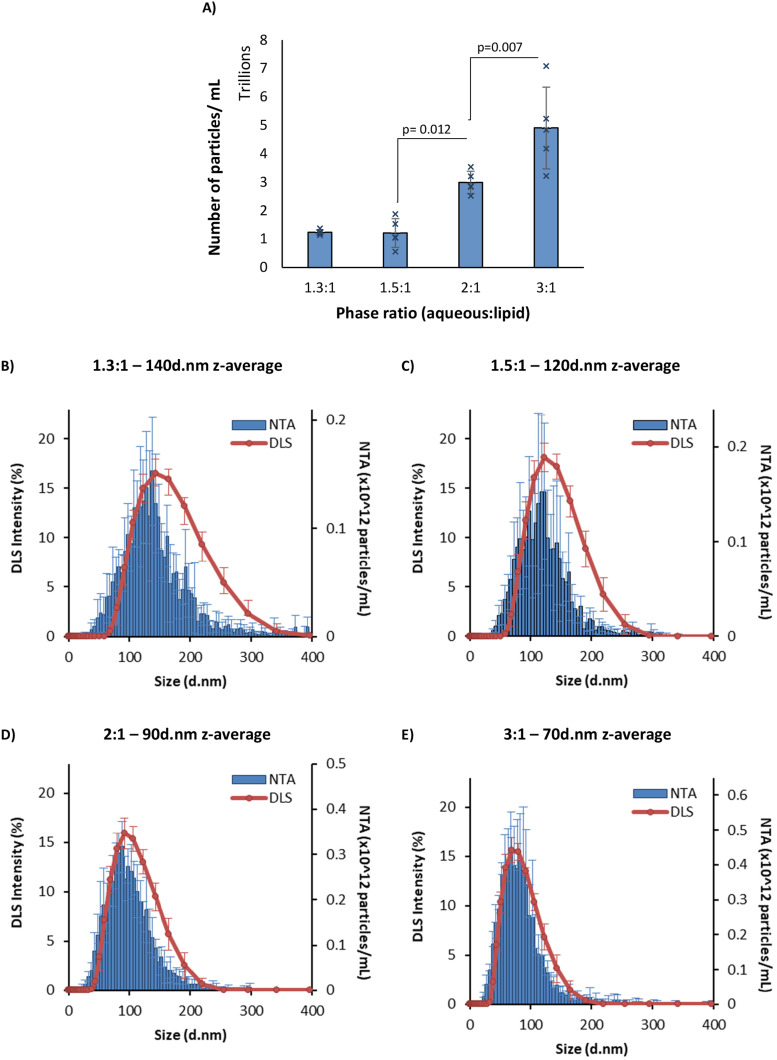
NTA *vs.* DLS data. (A) Number of particles per mL in 1 mg mL^−1^ LNP formulation. (B–E) NTA concentration *vs.* DLS intensity plots at four different aqueous : organic phase ratios. NTA data (blue) and DLS traces (orange). The *z*-average is the intensity mean given by the DLS for each sample. The LNPs were manufactured with an N/P ratio of 6 from 5 mg mL^−1^ lipid stock and purified by dialysis in pH 7.4 PBS for 1 hour. Each measurement is a mean of three independent batches with error bars showing ±SD. The significance is indicated by an asterisk (*) between each of the phase ratios analysed by one-way ANOVA with Tukey's pairwise comparisons. NTA = nanoparticle tracking analysis; DLS = dynamic light scattering.

The size of different phase ratios was also measured using nanoparticle tracking analysis (Fig. 2). This technique allowed for the number of particles to be estimated in each sample (Fig. 2A), showing per 1 mg mL^−1^ lipid concentration there were more particles for smaller LNPs and fewer particles of the larger LNPs, which makes sense, due to the same amount of material being present in each sample. All of the samples tested had a similar span of between 0.9 and 1.1, indicating the same particle size distribution in the formulations tested. Interestingly, the same number of particles was found in the 1.5 : 1 and 1.3 : 1 samples, despite a 20 nm difference in their *z*-average size, suggesting that this difference in particle size is not solely dependent on the number of lipid molecules within each LNP. Comparing the intensity-to-frequency plots (Fig. 2B–E) showed a similar size distribution given by both techniques. The smaller particles (2 : 1 and 3 : 1, 90 d.nm and 70 d.nm) map (Fig. 2D and E), whereas the larger particles (1.3 : 1 and 1.5 : 1, 140 d.nm and 120 d.nm) show a shift in the DLS intensity readings towards higher particle size. This is likely due to larger particles contributing more to the overall intensity in scattered light for DLS measurements,^15,16^ resulting in a greater intensity value in the intensity distributions shown. Overall, these data confirm the ability to precisely control LNP size through adjustments in the aqueous-to-organic phase ratio for both ALC-0315 and SM-102 ionisable lipid-containing LNPs, as previously seen with liposomes and LNPs.^3,7,9,10,17^

**Fig. 3:**
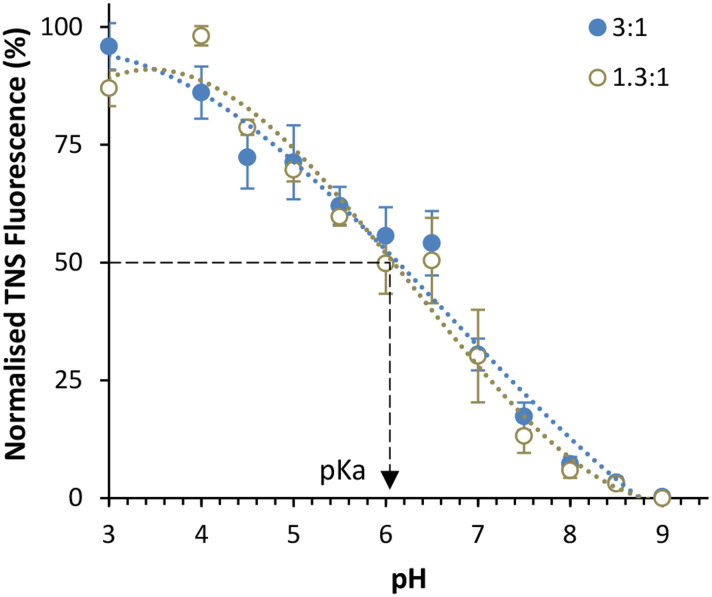
Measuring the p*K*_a_ of ALC-0315 ionisable lipid LNPs. The normalised fluorescence values of TNS when incubated with ALC-0315 LNPs titrated in buffers over a pH range of 3–9. Citrate buffer was used for pH 3–6, sodium phosphate buffer was used for pH 6.5–8, and Tris buffer was used for pH 8.5–9. The readings were normalised per each maximum value for each LNP batch. Each data set is representative of 3 batches of LNPs manufactured at an aqueous : lipid phase ratio of 3 : 1 (blue solid circles) or 1.3 : 1 (grey open circles). The p*K*_a_ value was found by taking the pH value at 50% of the maximum fluorescence. To fit a sigmoidal curve to the data, polynomial lines of best fit to the order of 3 were used. The LNPs were manufactured at an FRR of 3 : 1 with an N/P ratio of 6 from 5 mg mL^−1^ lipid stock, and pH 7.4 PBS was used as the exchange buffer.^8^

**Fig. 4:**
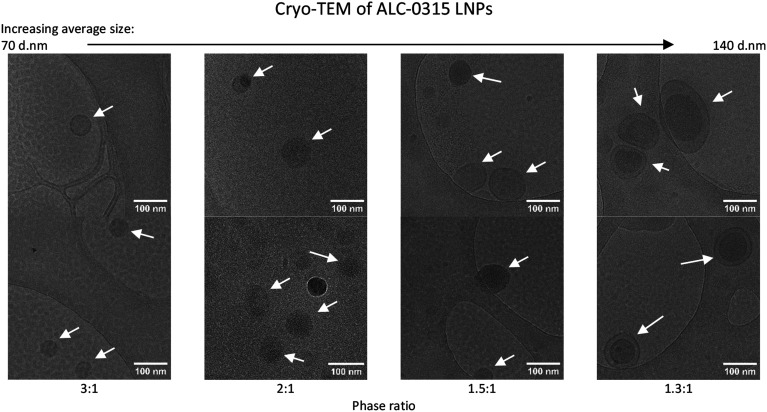
Cryogenic transmission electron microscopy of LNPs with ALC-0315 as the ionisable lipid, manufactured at four different aqueous : lipid phase ratios. There are two images at 30 000× magnification for each manufacturing phase ratio, and LNPs are shown by white arrows. The LNPs were manufactured with an N/P ratio of 6 from 5 mg mL^−1^ lipid stock. pH 7.4 PBS was used as the exchange buffer, and they were maintained in this buffer at manufacturing concentrations when cryogenically frozen in liquid ethane and imaged in liquid nitrogen using a Jeol JEM-F-200 microscope at 200 kV.

To further investigate LNPs made at different sizes, the corresponding p*K*_a_ values for these LNPs were measured using the TNS assay (Fig. 3) in a method adapted from previous studies.^11,13^ The p*K*_a_ values of LNPs generally fall within the range of 6–7.^18,19^ Using the Henderson–Hasselbalch equation, this signifies that under pH conditions equal to those of the p*K*_a_, the ionisable lipid is 50% protonated, while at one pH unit below the p*K*_a_ it is 90% protonated, and at two units it is 99% protonated. This indicates that at a manufacturing pH of 4, the ionisable lipid is almost entirely cationic, and therefore, this has been used as a reliable manufacturing pH for LNPs in research and commercially.^20,21^ The TNS fluorescence is a direct indicator of the % protonation of the ionizable lipid. Upon fixing the pH at which the TNS emits 50% of the maximum fluorescence in the presence of the ALC-0315 LNPs, the p*K*_a_ value can be estimated. For both small (phase ratio = 3 : 1) and large (phase ratio = 1.3 : 1) LNPs, the p*K*_a_ was approximately 6.1, in agreement with prior literature^18,19^ and confirms that the lipid is sufficiently protonated under manufacturing conditions (pH 4) for full mRNA encapsulation. We also observe that the manufacturing phase ratio and size of the LNPs have no impact on the p*K*_a_ and so this variable should not impact the mRNA encapsulation and expressing potential of the LNPs. To examine any potential physical differences between the different-sized LNPs, cryo-TEM was performed to image each phase ratio of the LNPs (Fig. 4), and this presented similar filled-circular structures for the first three smaller ratios. However, at the 1.3 : 1 phase ratio, a double-lamellar LNP was found to be the most-commonly presented structure. This shows that there are structural differences between this size of LNP and that of the smaller LNPs, and that these are perhaps necessary to form a stable lipid nanoparticle of this size. Furthermore, as stated, during the formation of these LNPs where the ethanol concentration is higher, there is more opportunity for lipids to aggregate and form larger LNPs. This may result in different morphological structures with the potential for phase separation of the dense mRNA and ionisable lipid core, leaving the other lipids to form extra layers, similar to what has been observed in the formation of bleb-rich LNPs.^22^ Furthermore, this double-layer morphology has previously been observed in larger LNPs and liposomes.^3,23^

### 3.2. The impact of aqueous : organic phase ratio and LNP size on *in vitro* and *in vivo* expression

**Fig. 5:**
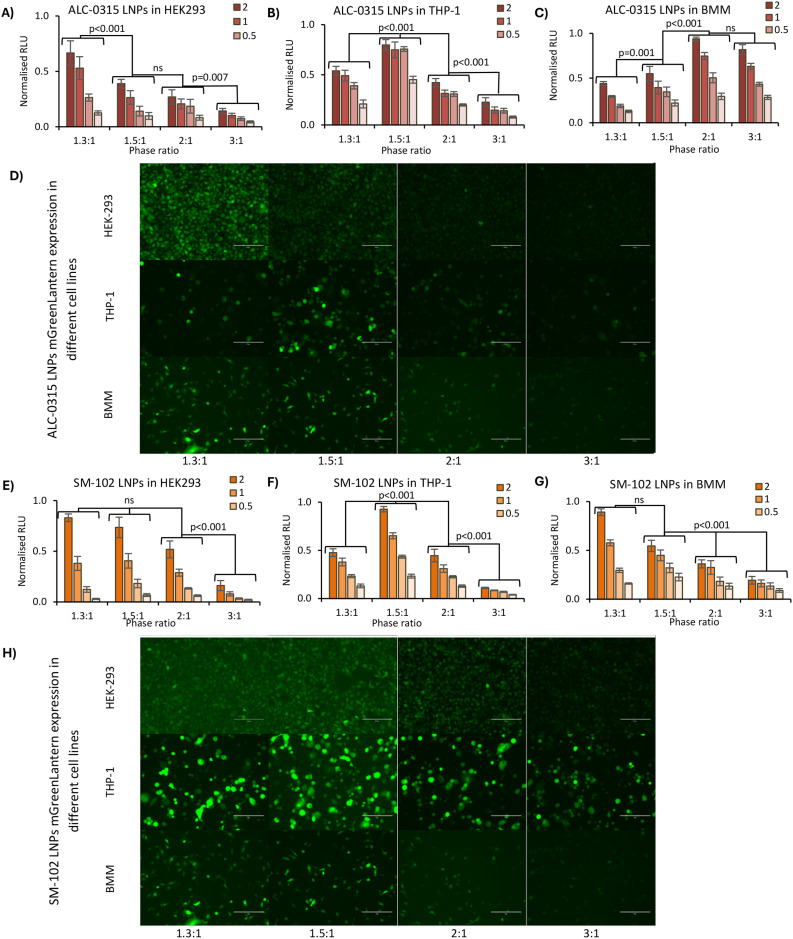
*In vitro* expression of LNPs in HEK-293, THP-1 and BMM (bone marrow-derived macrophages) cells. (A–C) FLuc mRNA expression of ALC-0315 LNPs in HEK-293 (A), THP-1 (B) and BMM (C) cells. (D) Images showing mGreenLantern expression of ALC-0315 LNPs in HEK-293, THP-1 and BMM cells. (E–G) FLuc mRNA expression of SM-102 LNPs in HEK-293 (E), THP-1 (F) and BMM (G) cells. (H) Images showing mGreenLantern expression of SM-102 LNPs in HEK-293, THP-1 and BMM cells. For the luciferase expression (A–C, E–G), each formulation was added to the cells at four concentrations of mRNA shown as dark to light coloured bars as the concentration decreases from 2 μg mL^−1^, 1 μg mL^−1^, and 0.5 μg mL^−1^ to 0.25 μg mL^−1^. The expression is shown as normalised relative light units (RLU), taking the maximum measurement from each plate reading and dividing all the values by this maximum. The LNPs were manufactured with an N/P ratio of 6 with either FLuc mRNA (A–C, E–G) or mGreenLantern mRNA (D and H) from 5 mg mL^−1^ lipid stock and purified by dialysis in pH 7.4 PBS for 1 hour. For the graphs, each measurement is a mean of three independent batches with error bars showing ±SEM. The significance is indicated by *p* values obtained by two-way ANOVA for individual treatment group comparisons.

**Fig. 6:**
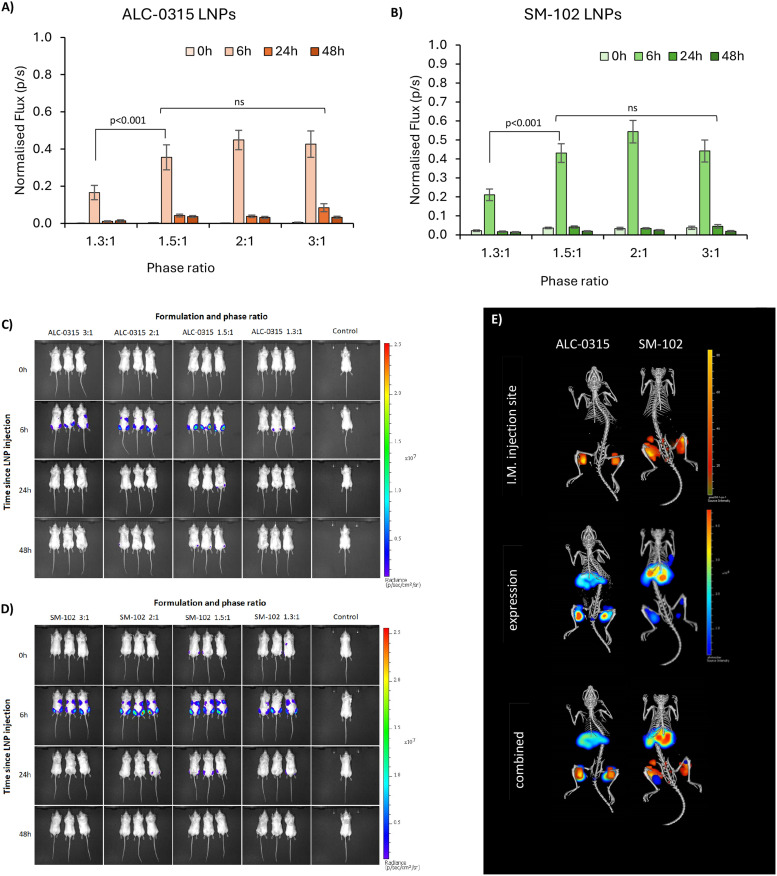
Biodistribution and expression of ALC-0315 and SM-102 LNPs following I.M. injection in BALB/C mice. (A and B) Expression is shown as normalised bioluminescence at four time points after injection – 0 (10 minutes), 6, 12 and 24 hours (light to dark bars) in ALC-0315 LNPs (A) and SM-102 LNPs (B). Error bars are representative of SEM. The significance is indicated by an asterisk (*) between each of the phase ratios analysed by three-way ANOVA with Tukey's pairwise comparisons, and two-way ANOVA for individual treatment group comparisons. (C) Bioluminescence – mRNA expression images in mice injected with ALC-0315 LNPs. (D) Bioluminescence – mRNA expression images in mice injected with SM-102 LNPs. (E) CT scans of one mouse from each group, showing the 3D expression and intramuscular (injection site) biodistribution of the LNPs. Images C–D are one of 3 repeats, representative of the overall results.

As the aqueous : organic phase ratio reliably and reproducibly enabled the control of LNP size, this was then used to infer the impact of LNP size on expression, both *in vitro* and *in vivo*. LNPs encapsulating firefly luciferase mRNA were prepared, which when expressed catalyses luciferin into a bioluminescent product. The LNPs were added to HEK293, THP-1 and BMM cells, as three distinct cell lines at four standard concentrations in the 0.25–2 mg mL^−1^ test concentration range.^24–26^We observed that in HEK-293 cells the largest LNPs resulted in a 4 times higher expression than the smallest LNPs (Fig. 5A and E), with a pattern of gradually decreasing comparative expression as the phase ratio increases (and size decreases). This was true for both ALC-0315 and SM-102 LNPs adjusted to four different concentrations of mRNA. This same trend of higher expression for larger LNPs *versus* standard-size LNPs has previously been observed in LNPs manufactured with Dlin-MC3-DMA as the ionizable lipid in NCI-H358 cells.^10^ Larger LNPs were more likely to be internalised by cells *via* macropinocytosis instead of receptor-dependent endocytosis and it was discussed how larger particles encapsulate higher mRNA content, which is supported by our findings (Fig. 2A), showing fewer particles with larger-sized LNPs, whilst containing the same amount of mRNA within the sample. A reason why fewer particles with higher mRNA content may be more efficient for mRNA delivery and expression than more small particles containing less mRNA may be due to limited cell uptake. Though the uptake of labelled mRNA was found to be the same for each LNP size, expression differences were found to be independent of internalization, perhaps owing to the differing effects of LNP composition and internalisation mechanisms on downstream intracellular processes. In THP-1 cells, a similar pattern was observed, except for slightly lower expression in the 1.3 : 1 phase ratio samples (Fig. 5B and F), which may be due to less uptake of these larger LNPs into this cell type. For BMM cells, a differing trend was seen between ALC-0315 and SM-102 manufactured LNPs (Fig. 5C and G); the SM-102 samples exhibited the same preference for larger particles as shown in the other cell lines, but the ALC-0315 data showed an opposite trend. It is unknown as to why the expression pattern is different in this cell line, specifically for one ionisable lipid, where the LNPs manufactured with different ionisable lipids have previously shown the same pattern. This shows that the trend of larger LNPs expressing more is true for most, but not all formulations of LNPs in the tested cell lines. To visually observe differences in mRNA expression in these three cell lines, LNPs were manufactured with mGreenLantern mRNA for capturing images of this green fluorescent protein when expressed in cells (Fig. 5D and H); these showed the same expression patterns as the FLuc mRNA LNPs, with the exception being the ALC-0315 LNPs in BMM again (quantified in Fig. S1†). This is observed in Fig. 6E, showing a CT scan and 3D biodistribution and expression of ALC-0315 and SM-102 LNPs. Furthermore, contrary to most of the *in vitro* results, the largest LNPs had the lowest expression *in vivo*, while the other sizes showed no significant difference in expression, which was found to be variable (Fig. 6). These largest LNPs manufactured at a phase ratio of 1.3 : 1 have notably different morphology with potential phase separation of the lipids and this, combined with their larger size, could be impacting on *in vivo* performance as a result of different biological interactions and/or stability. This lack of correlation between *in vitro* and *in vivo* results with respect to LNPs and other nanomedicines is not uncommon,^27–30^ as there are many impacting factors *in vivo*, such as biological milieu interactions,^31^ immune system interactions,^32^ circulation and movement to target site amongst others. This presents major challenges in screening LNPs and the down-selection from *in vitro* to *in vivo* studies. Furthermore, homogeneous cell cultures do not represent the heterogeneous complexity of a living organism, and the different pharmacokinetic barriers, which could impact the stability and potency of different formulations. This indicates major limitations in relying on these *in vitro* models to predict *in vivo* outcomes and highlights the importance of *in vivo* testing. However, there is also potential for discrepancies between preclinical trials in murine models and clinical outcomes, and therefore additional models are required to better predict clinical outcomes. Typically the optimal size for LNPs is in the 50–100 nm size range depending on the formulation,^3,33^ and previous studies have suggested that smaller LNPs may be better indicated for siRNA-based therapies, and larger LNPs for those that are mRNA-based, in conjunction with the molecular sizes of the nucleic acid cargo,^10^ so it may be that larger sizes can more efficiently pack mRNA, but smaller LNPs are more stable, creating an optimal range for LNP expression. Our data suggest that LNPs between approximately 60 and 120 d.nm have equivalent mRNA expression after intramuscular injection. This suggests that particle size is a key critical quality attribute and is sensitive to small changes in the microfluidic mixing processes; however, it is less influential on potency in small-animal studies. Indeed, this has also been shown in mRNA vaccine studies as Hassett *et al.*^3^ looked at immune responses in mice and non-human primates, where all tested sizes of SM-102 ionisable lipid LNPs from 60–150 d.nm gave consistent results. This study supports and further investigates this work by specifically looking at mRNA expression in *in vitro* and *in vivo* across the same size range in both SM-102 and ALC-0315 LNPs.

## 4. Conclusions

In this study, the impact of the aqueous : organic buffer phase ratio was evaluated for its ability to alter ALC-0315- and SM-102-formulated LNP critical quality attributes. We observed that the phase ratio correlated with LNP size leading to the manufacture of LNPs with differing sizes ranging from 60 to 140 nm in diameter for further evaluation. LNPs over the size of 120 d.nm showed a different morphology to smaller LNPs observed using CryoTEM, with a double-lamellar structure being the most common. Particle size is a well-recognised critical quality attribute for LNPs and critical to the quality and efficacy of an LNP product. Generally, LNPs used in the clinic are in the 50–100 nm size range and manufactured at a phase ratio of 3 : 1. The manufacture of smaller size ranges and larger LNPs can be used to infer a more detailed view of the impact LNP particle size has on expression. Our *in vitro* experiments in three distinct cell lines revealed that larger LNPs usually resulted in higher mRNA expression than smaller sized LNPs. This has been attributed to different uptake mechanisms and the impact on downstream intracellular processes. However, when tested *in vivo*, this was no longer the case. *In vivo*, we saw the largest LNPs give the lowest expression, and the other sizes were not significantly different from each other.

Here we show that small changes in manufacturing will impact LNP particle size, but this does not necessarily translate to changes in their efficacy. Furthermore, the issue regarding the lack of correlation between *in vitro* and *in vivo* results elucidates that although *in vitro* studies are fundamental for assessing toxicity and baseline expression of formulations, *in vivo* studies are crucial when evaluating the efficacy of formulations.

## Author contributions

Conceptualization, methodology, formal analysis, investigation, data curation, writing – original draft preparation, writing – reviewing and editing, visualisation, CMcM, AD, SR, AB, BB, ZR, YP; supervision, AD, SR, ZR, YP; project administration, funding acquisition, AD, SR, YP. All authors have read and agreed to the published version of the manuscript.

## Data availability

The supporting data set is available online at https://doi.org/10.15129/fb395621-acf1-4edc-aab0-fc5275d48bdf.

## Conflicts of interest

There are no conflicts to declare.

## Supplementary Material

PM-001-D4PM00128A-s001
