# Supplementary material for: Tailoring lipid nanoparticle dimensions through manufacturing processes
Source: RSC Pharm. 2024 Sep 23;1(4):841–53. doi: 10.1039/d4pm00128a (PMC11417672; doi:10.1039/d4pm00128a)
Supplement: PM-001-D4PM00128A-s001 [file PM-001-D4PM00128A-s001.pdf]

Supplementary Information (SI) for RSC Pharmaceutics.
This journal is © The Royal Society of Chemistry 2024

Supplementary information

Table S1: NTA data particle trajectories (tracks).

**Aqueous: organic phase ratio**	**Tracks per capture number**					**Total tracks**	**Valid tracks (total particle count)**
	1	2	3	4	5		
1.3:1	2773	1676	1493	1515	1731	9188	**2774**
1.5:1	1906	1779	1809	1533	1495	8522	**3623**
2:1	6770	3570	4048	6766	2493	23647	**4976**
3:1	3207	3984	6882	3131	2926	20130	**5056**

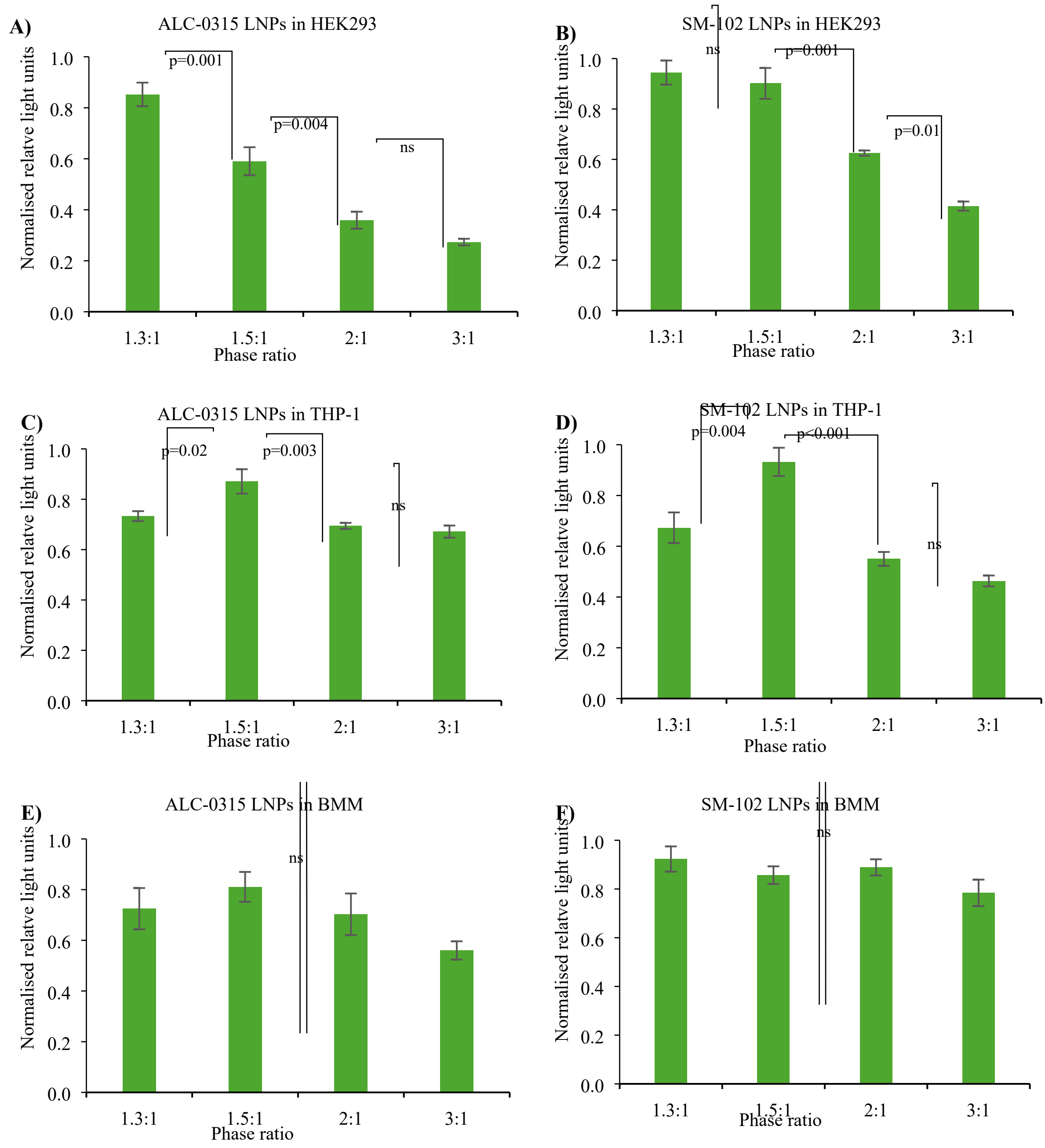

Figure S1: GFP fluorescence quantification from mGreenLantern-loaded LNPs. Comparing the different aqueous:lipid phase ratios in HEK-293 (A-B), THP-1 (C-D) and BMM (E-F) cells for LNPs manufactured with ALC-0315 (A, C, E) and SM-102 (B, D, F) as the ionisable lipid. Values quantified using ImageJ, as an average of 5 images from each sample. Significance values shown calculated from one-way ANOVA.

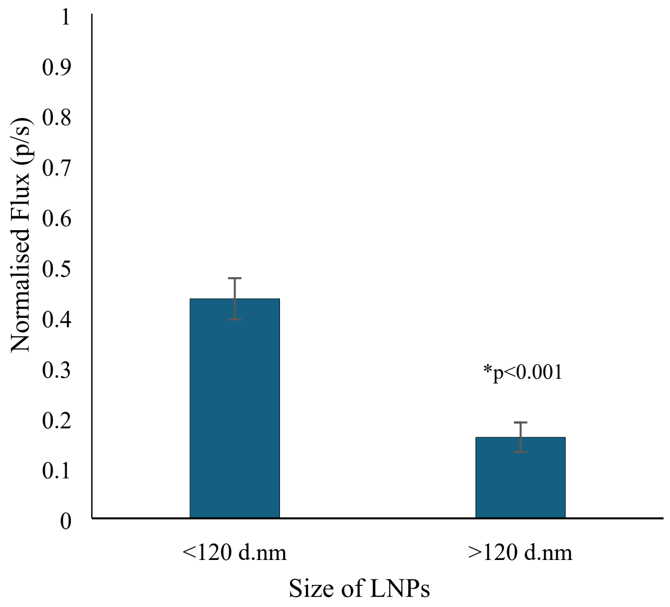

Figure S2: In vivo size versus mRNA expression of LNPs. Comparing LNPs manufactured at sizes smaller than 120d.nm (left) and larger than 120d.nm (right). Error bars shown as ±S.E.M. Statistically analysed using a two-sample unpaired T-test.
